# Associations among neurophysiology measures in irritable bowel syndrome (IBS) and their relevance for IBS symptoms

**DOI:** 10.1038/s41598-020-66558-w

**Published:** 2020-06-17

**Authors:** Irina Midenfjord, Annikka Polster, Henrik Sjövall, Peter Friberg, Hans Törnblom, Magnus Simrén

**Affiliations:** 1 Department of Internal Medicine and Clinical Nutrition, Institute of Medicine, Sahlgrenska Academy, University of Gothenburg, Gothenburg, Sweden; 2 Department of Molecular and Clinical Medicine, Sahlgrenska Academy at Gothenburg University, Gothenburg, Sweden; 3 Centre for Functional GI and Motility Disorders, University of North Carolina, Chapel Hill, NC United States

**Keywords:** Gastroenterology, Gastrointestinal diseases

## Abstract

Abnormal gut-brain interactions are common in irritable bowel syndrome (IBS), but the associations between neurophysiological measures and their relation to gastrointestinal (GI) symptoms are poorly understood. Our aim was to explore these relationships and define the most relevant neurophysiology measures for GI symptom severity in IBS. IBS patients underwent small intestinal motility (manometry; fasted and fed contraction frequency, phase III time) and secretion (transmural potential difference), rectal sensorimotor (barostat; sensory thresholds, tone response, compliance), autonomic nervous system (baroreceptor sensitivity and effectiveness), and colonic motor function (transit time) examinations. GI symptom severity (GSRS-IBS), and anxiety and depression (HAD) as a proxy measure of central nervous system (CNS) dysfunction, were assessed. In total 281 IBS patients (Rome II criteria) were included (74% females, median age 36 [interquartile range 28–50] years). Significant correlations between neurophysiology measures were stronger within, rather than between, different neurophysiological examinations. The strongest neurophysiology-symptom correlations occurred between a combination of CNS and visceral sensitivity parameters, and GSRS-IBS total score and pain domain (ρ = 0.40, p < 0.001, and ρ = 0.38, p < 0.001). Associations between GI symptoms in IBS and individual and combinations of neurophysiological factors occurred, primarily in CNS and visceral sensitivity measures, providing new insights into the clinical presentation of IBS.

## Introduction

Irritable bowel syndrome (IBS) is a common and complex functional gastrointestinal (GI) disorder where gut-brain interactions^1,2^, and alterations in the gut microenvironment^3^ are considered to be central in the pathophysiology. Abdominal pain, related to defecation and associated with changes in stool form or frequency, are the characteristic clinical features of this female predominant disease^1^ with 5–10% prevalence worldwide^4–6^. The disease leads to high costs for society, due to increased use of health care services^7^, as well as lowered work productivity and higher absenteeism from work^8,9^.

Different abnormalities involved in gut-brain interactions are present in IBS, leading to a complex clinical presentation, but to date the pathophysiology is not completely understood. Various pathophysiological factors have been brought forward as important for symptom generation in IBS, but none of these is present in all patients with IBS. IBS patients have been reported to have increased psychological distress^10,11^, visceral hypersensitivity^12^, altered colonic motility^13^, aberrant autonomic nervous system (ANS) function^14,15^, rectal sensorimotor dysfunction^16,17^, and dysfunction of motility^18–20^ and secretion^21^ of the small intestine, in comparison with healthy controls. Although these abnormalities have been described individually in IBS, the associations among these aberrant measures, and the interactions between these parameters and the patient reported IBS symptom severity, have to this date not been thoroughly studied. Associations between overall IBS symptom severity and psychiatric comorbidities^22,23^ or visceral hypersensitivity^12,17,24–26^ have been previously demonstrated. The link between overall IBS symptom severity and altered colonic motility is less obvious, as associations with abnormal bowel habits, but not other GI symptoms, have been the most commonly reported finding^13,27^. However, for the other neurophysiological factors, the association with symptom severity in IBS is even less clear.

Since the interaction among the different neurophysiological alterations demonstrated in IBS is incompletely understood, achieving a better understanding of the interactions between the reported symptoms and the complex clinical presentation of IBS is of importance to improve the clinical management of these patients. In line with the concept that IBS is a disorder of gut-brain interactions^1,2^, associations between the patient reported GI symptom severity and individual neurophysiological factors involved in gut-brain interactions were assessed. Specifically, we included psychological distress as a proxy measure for CNS dysfunction^11,28^, rectal sensorimotor function, small intestinal motility, small intestinal secretion, colonic motility, and baroreceptor (BR) sensitivity as a proxy for ANS function^15,29^, or combinations thereof, in a large group of well-characterized IBS patients in multivariable analyses. The aim of this study was to explore the relationships among these factors and to extract the most important variables for symptom generation in IBS, in order to expand our understanding of the complex clinical presentation of IBS.

## Results

### Descriptives

The total cohort of IBS patients (N = 281) consisted of 74% females, and had a median age of 36 years, as shown in Table 1. IBS-D was the most common IBS subtype (43%), followed by IBS-A (32%) and IBS-C (25%). The median anxiety and depression levels of the patient cohort were in the normal range^30^ and did not differ between subgroups, and the median scores of the GSRS-IBS domains were mostly in the range of moderate symptom severity^31^. The median values of the 16 neurophysiological factors used in this study are presented in Table 2. Only few and predominantly expected differences were seen between IBS subgroups; severity of diarrhoea and constipation, colonic transit time, total phase III time in the small bowel, and the mean value of the transmural potential difference in the small intestine. Hence, no division of patients into IBS subgroups were made for further analyses.

**Table 1:** Characteristics of the IBS-patients.

Characteristic	IBS patients (N = 281)	IBS-C(N = 55)	IBS-D(N = 95)	IBS-A(N = 70)	p-value
Age in years	36 [28–50]	36 [29–49]	41 [28–53]	35 [27–50]	0.48
Female sex	74%	78%	73%	76%	1.0*
IBS subtypes					
IBS-C	25%				
IBS-D	43%				
IBS-A	32%				
GSRS-IBS					
Pain syndrome	4 [3–5]	4 [3–5]	4 [4–5]	4 [3–5]	0.15
Bloating syndrome	5 [3–6]	5 [4–6]	5 [3–6]	5 [4–6]	0.31
Constipation syndrome	3 [1–4]	4 [3–5]	2 [1–3]	3 [1–4]	**<0.001**
Diarrhoea syndrome	3 [3–5]	3 [3–4]	4 [3–5]	3 [2–4]	**0.003**
Satiety syndrome	2 [1–3]	2 [1–3]	3 [2,3]	2 [1–3]	0.28
Total	3.5 [2.8–4.2]	3.7 [3.1–4.4]	3.7 [2.9–4.2]	3.2 [2.7–4.1]	0.07
Anxiety (HAD-A)	6 [4–10]	6 [4–10]	6 [4–12]	6 [4–10]	0.68
Depression (HAD-D)	4 [2–7]	4 [2–8]	5 [2–8]	4 [2–6]	0.31

Presented as proportions (%) or as median with interquartile range. Differences between medians in the subgroups were analysed by Kruskal-Wallis tests or Chi squared test (marked with *). Significant p-values (p < 0.05) are marked in bold.

IBS: Irritable bowel syndrome; IBS-A: alternating IBS; IBS-C: constipation predominant IBS; IBS-D: diarrhoea predominant IBS; HAD-A: Hospital anxiety and depression scale, anxiety subscale; HAD-D: Hospital anxiety and depression scale, depression subscale.

**Table 2:** Median values of the neurophysiological measures used in this study.

Neurophysiological factor	Whole cohort (N = 281)	IBS-C (N = 55)	IBS-D (N = 95)	IBS-A (N = 70)	p-value
Central nervous system (N = 266)					
Psychological distress(HAD total score)	11 [6–16]	10 [7–16]	12 [9–16]	12 [6–16]	0.58
Colonic motility (N = 210)					
Colonic transit time(days)	1.4 [1.0–2.1]	2.1 [1.3–3.5]	1.1 [0.7–1.7]	1.5 [1.1–2.1]	**<0.001**
Rectal sensorimotor (N = 205)					
Early rectal tone response(%)	31 [1.5–63]	33 [7.1–57]	30 [0.8–62]	26 [−2.7–63]	0.94
Late rectal tone response(%)	36 [1.1–80]	35 [10–79]	25 [−15–75]	25 [−7.9–71]	0.57
Rectal dynamic compliance(ml mmHg^−1^)	5.9 [4.2–8.2]	5.9 [4.9–8.2]	6.3 [4.1–8.5]	6.2 [3.7–8.0]	0.83
Rectal static compliance(ml mmHg^−1^)	8.4 [6.2–10]	9.0 [6.6–10.4]	7.9 [5.9–9.6]	8.3 [6.1–10]	0.60
Rectal first sensation threshold(mmHg)	7 [7–12]	7 [7–12]	7 [7–12]	7 [7]	0.07
Rectal pain threshold(mmHg)	32 [27–42]	32 [26–45]	31 [22–39]	32 [27–42]	0.20
Small intestinal motility (N = 130)					
SI phase III time(s)	477 [356–776]	412 [358–694]	659 [434–803]	414 [305–569]	**0.003**
SI fasted contraction frequency(minute^−1^)	1.4 [1.1–1.9]	1.4 [1.2–1.8]	1.7 [1.3–2.0]	1.4 [1.1–1.6]	0.06
SI fed contraction frequency(minute^−1^)	2.9 [2.4–3.4]	3.1 [2.6–3.4]	2.8 [2.4–3.5]	2.7 [2.3–3.5]	0.64
Small intestinal secretion (N = 130)					
SI potential difference, mean(mV)	−0.31 [−0.77–0.23]	−0.31 [−0.85–0.06]	−0.59 [−0.98–0.01]	−0.02 [−0.40–0.63]	**0.01**
SI potential difference, max(mV)	−9.9 [−11–8.8]	−9.6 [−10–9.3]	−10 [−11–8.9]	−9.8 [−11–8.1]	0.70
SI potential difference, rate of rise (mV s^−1^)	−0.065 [−0.095–0.048]	−0.09 [−0.19–0.06]	−0.06 [−0.08–0.04]	−0.07 [−0.09–0.05]	0.06
Autonomic nervous system (N = 87)					
Baroreceptor sensitivity(ms mmHg^−1^)	14 [10–19]	11 [8–18]	14 [10–19]	13 [11–18]	0.83
Baroreceptor effectiveness	0.36 [0.22–0.53]	0.28 [0.22–0.50]	0.35 [0.24–0.48]	0.47 [0.19–0.57]	0.54

Presented as medians with interquartile ranges. Number of patients completing the different neurophysiologic examinations are described in each heading. Differences between medians in the subgroups were analysed by Kruskal-Wallis tests. Significant p-values (p < 0.05) are marked in bold.

HAD: Hospital Anxiety and Depression scale; IQR: interquartile range; SI: small intestine/intestinal.

### Correlations among neurophysiological factors and IBS symptoms

**Figure 1:**
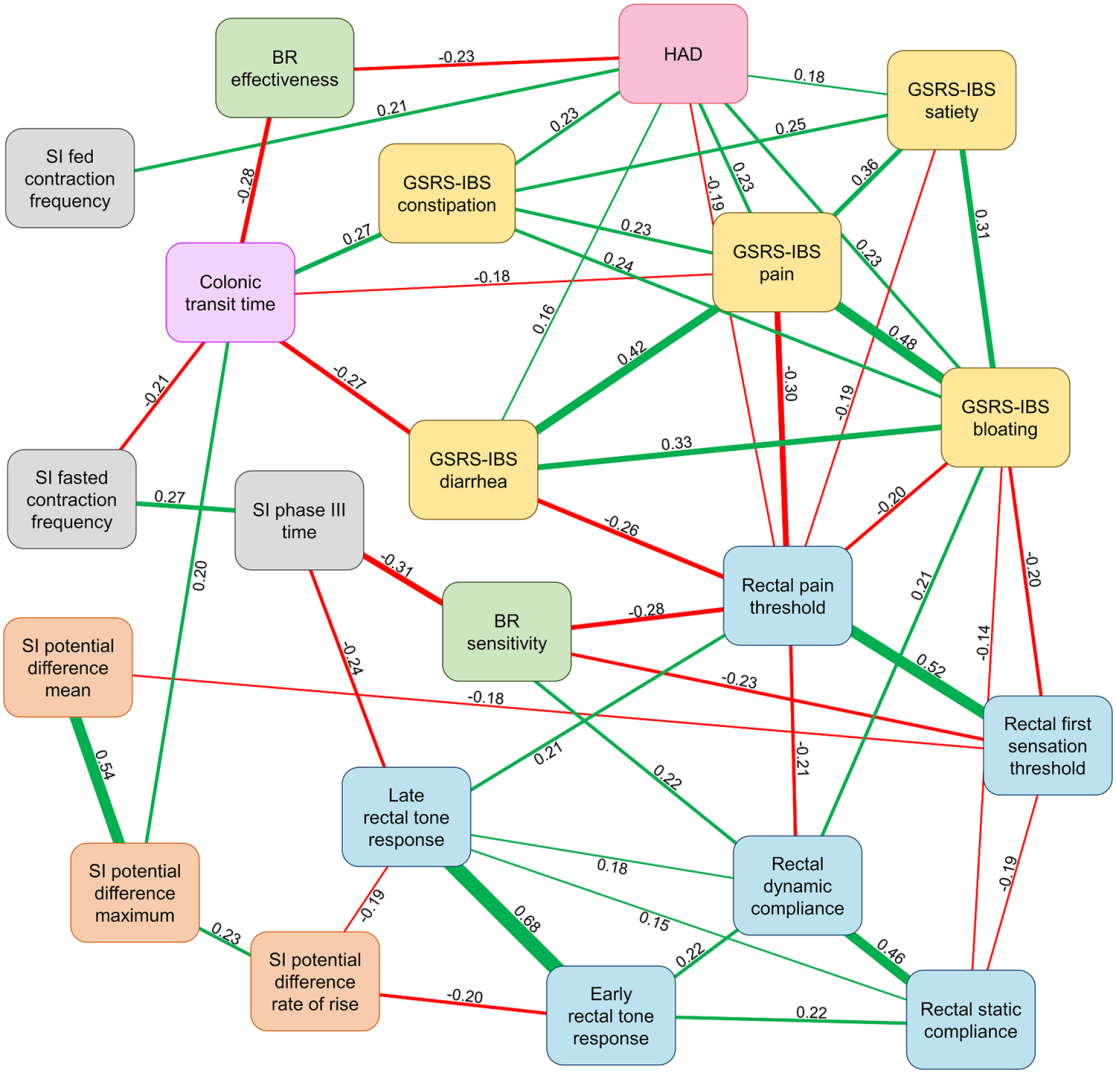
Correlations between the neurophysiological factors from the overall cohort of IBS patients (N = 281). Significant correlations between neurophysiological factors are shown in the figure (p < 0.05, two-tailed, unadjusted for multiple comparisons). Numbers represent Spearman´s rho. Green edges show positive correlations, whereas red edges show negative correlations. BR: Baroreceptor; GSRS-IBS: Gastrointestinal Symptom Rating Scale, IBS version; HAD: Hospital Anxiety and Depression scale; SI: Small intestine/intestinal.

Correlations were calculated among the neurophysiological factors and IBS symptoms in the whole cohort (N = 281), as can be seen in Fig. 1. The strongest correlations were mainly seen between different measures within the same neurophysiological examinations, e.g. factors of rectal tone (ρ = 0.68), small intestinal secretion (ρ = 0.54), visceral sensitivity (ρ = 0.52) and rectal compliance (ρ = 0.46). Weaker, but significant (p < 0.05), correlations were noted between some of the different neurophysiological measurements with ρ-values in the range of 0.2 to 0.3. The measures with the highest number of associations were the GSRS-IBS bloating domain, the rectal pain threshold and psychological distress.

Regarding associations between GI symptom severity and individual neurophysiological measures, significant (p < 0.05) correlations were seen mainly with psychological distress and rectal sensitivity variables. No significant (p < 0.05) correlations were detected between GI symptoms and small intestinal motility, small intestinal secretion, rectal tone response or ANS function. The strongest correlations were seen between rectal sensitivity and GSRS-IBS pain and GSRS-IBS diarrhoea (ρ = 0.30 and ρ = 0.26, respectively), and colonic transit time and GSRS-IBS constipation and GSRS-IBS diarrhoea (ρ = 0.27 and ρ = −0.27, respectively). Psychological distress demonstrated modest associations with all GSRS-IBS domains, whereas moderately strong associations were seen between the different GSRS-IBS domains (ρ = 0.23–0.48).

### Multivariate analyses

All study participants who had completed GSRS-IBS, i.e. the dependent variables, were included in the multivariate analyses (N = 193). The 16 neurophysiological factors were first processed to form the overall neurophysiology score. The Lasso scores for the domains and total score of GSRS-IBS, respectively, were then calculated through a summation of the Lasso-extracted combination of variables from the overall neurophysiology score, as can be seen in Table 3.

**Table 3:** Neurophysiological factors in the models after Lasso regression.

Neurophysiological factor	GSRS-IBS					
	Pain	Bloating	Constipation	Diarrhoea	Satiety	Total
HAD	X	X	X	X	X	X
Colonic transit time			X		X	
Early rectal tone response						
Late rectal tone response						
Rectal dynamic compliance						
Rectal static compliance						
Rectal first sensation threshold			X			
Rectal pain threshold	X	X		X	X	X
SI phase III time			X			
SI fasted contraction frequency			X			
SI fed contraction frequency						
SI potential difference, mean		X	X		X	
SI potential difference, max			X			
SI potential difference, rate of rise				X		
Baroreceptor sensitivity		X	X			
Baroreceptor effectiveness						
RMSE of Lasso score	1.16	1.27	1.56	1.18	1.23	0.85
*RMSE of overall score*	*1.24*	*1.40*	*1.64*	*1.27*	*1.48*	*0.89*
Correlation with Lasso score	0.38	0.20	0.14	0.29	0.29	0.40
*Correlation with overall score*	*0.21*	*0.23*	*0.10*	*0.16*	*0.13*	*0.25*

Neurophysiological factors in the models after Lasso regression in GSRS-IBS total score and domain scores of GSRS-IBS. Lower RMSE indicates lower variability and thus better predictive ability of the model. Correlations are stated as Spearman’s rho. For comparison, the correlations with the overall neurophysiology score are included.

GSRS-IBS: Gastrointestinal Symptom rating scale, IBS version; HAD: Hospital Anxiety and Depression scale; Lasso: Least Absolute Selection and Shrinkage Operator regression; overall: Neurophysiology scores derived from all 16 neurophysiological factors; RMSE: Root Mean Square Error; pain threshold: pressure threshold for pain during the balloon distension test of rectal sensorimotor function; SI: small intestine/intestinal.

**Figure 2:**
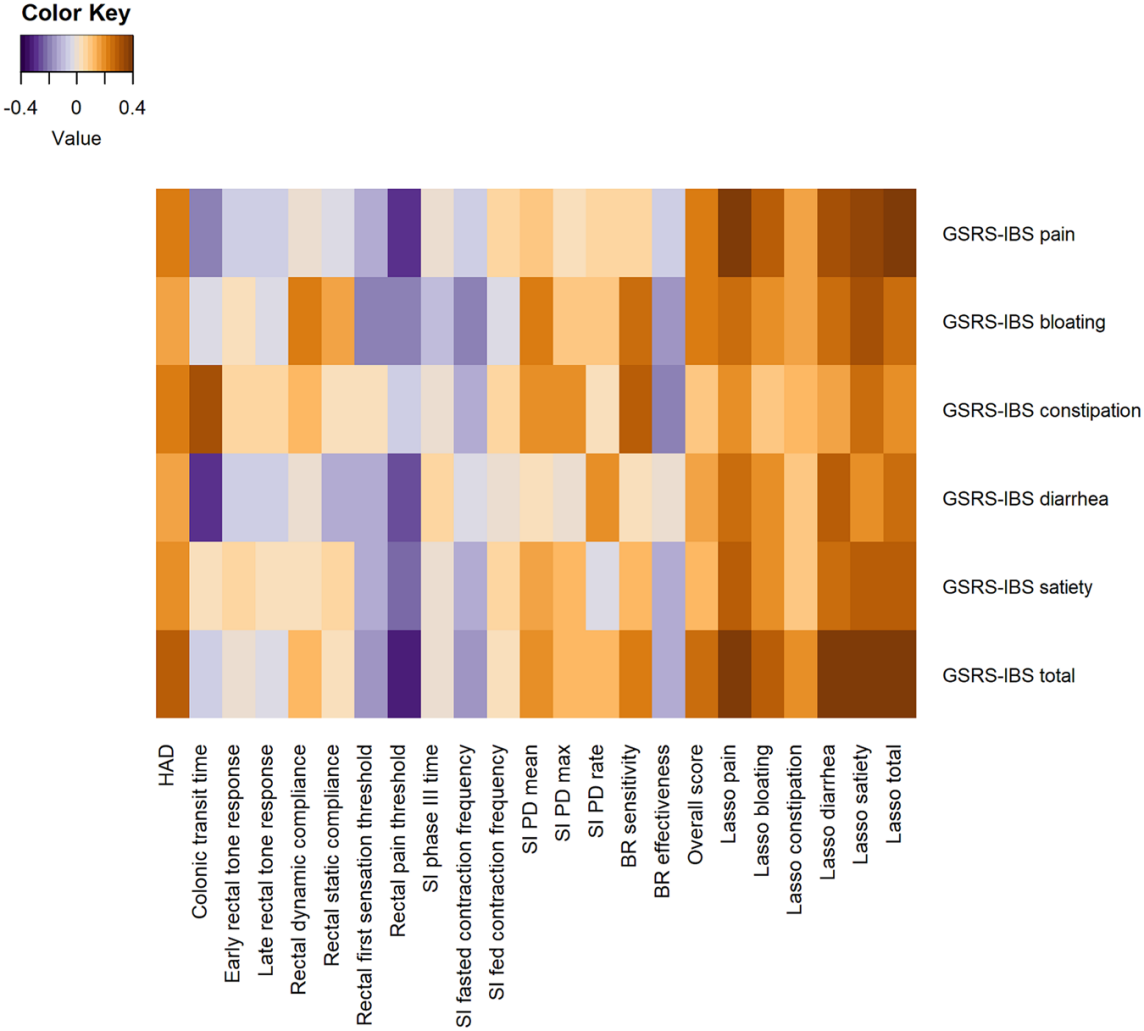
Heatmap of correlations in the overall cohort of IBS patients (N = 281), between GSRS-IBS total score and domain scores of GSRS-IBS, and single neurophysiological factors, the overall neurophysiology scores, or the Lasso scores, respectively. The specified correlation coefficients and p values are available in Supplementary Table 1. BR: baroreceptor; GSRS-IBS: Gastrointestinal Symptom Rating Scale, IBS version; HAD: Hospital Anxiety and Depression scale; Lasso: Least Absolute Selection and Shrinkage Operator regression; overall: Neurophysiology scores derived from all 16 neurophysiological factors; PD; potential difference; SI: small intestine/intestinal.

**Figure 3:**
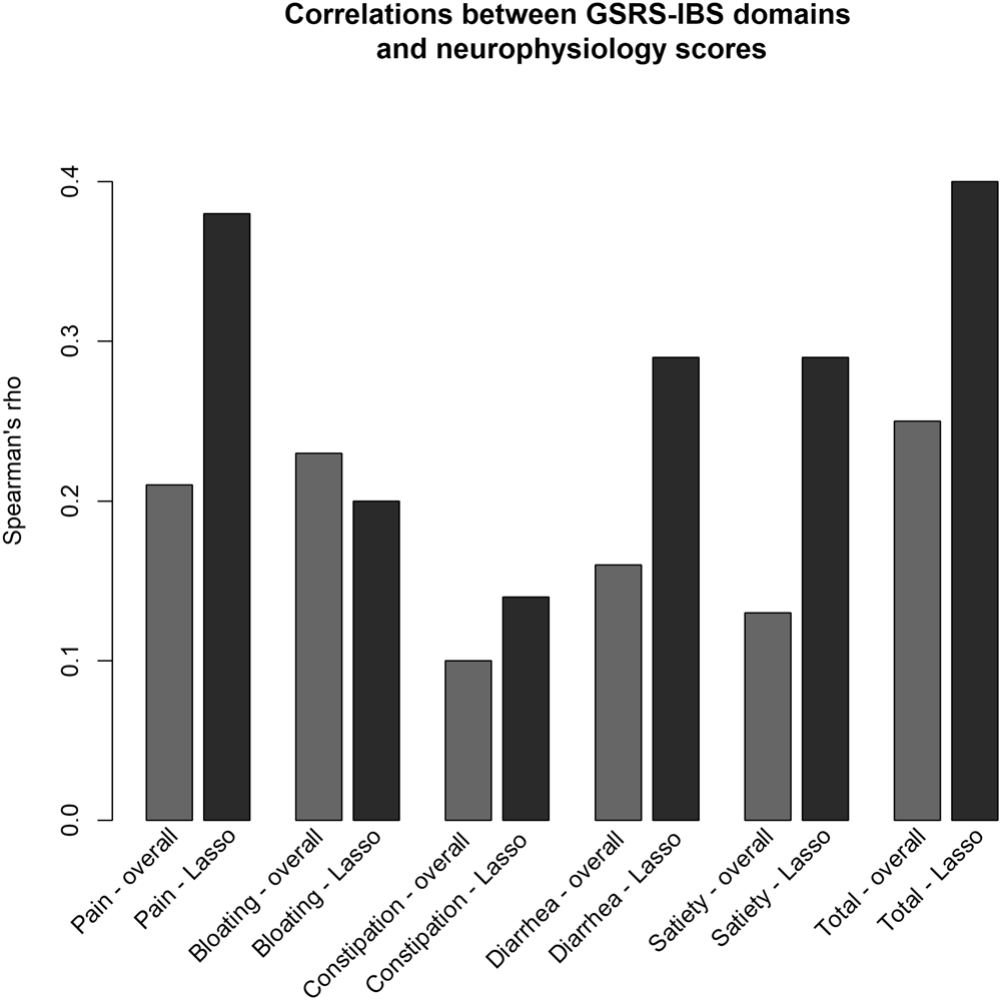
Comparisons of correlations between neurophysiology scores (overall neurophysiology score or Lasso scores) and GSRS-IBS total score and domain scores of GSRS-IBS in the overall cohort of IBS patients (N = 281). All correlations are significant (p < 0.05, two-tailed, false discovery rate adjusted for multiple comparisons), with the exceptions of Constipation - overall, Constipation – Lasso, Diarrhoea - overall and Satiety - overall. For specified p values, see Supplementary Table 1. GSRS-IBS: Gastrointestinal Symptom Rating Scale, IBS version; Lasso: Least Absolute Selection and Shrinkage Operator regression-derived neurophysiology scores; overall: Neurophysiology scores derived from all 16 neurophysiological factors.

A correlation heatmap was created to visualize the correlations between the GSRS-IBS total and domain scores of GSRS-IBS, and the single neurophysiological factors, as well as the overall neurophysiology score or Lasso scores (Fig. 2). The correlation coefficients and p values of the heatmap are presented in Supplementary Table 1. In general, most of the GSRS-IBS scores (total score and domain scores) had stronger correlations with the Lasso scores than with the overall neurophysiology score, although only moderately strong associations were detected (Fig. 3). The strongest correlation was found between GSRS-IBS total score and the corresponding Lasso score (ρ = 0.40, p < 0.001), which consisted of HAD and the rectal pain threshold (Table 3). The second strongest correlation was seen between GSRS-IBS pain and the corresponding Lasso score (ρ = 0.38, p < 0.001), which consisted of the same neurophysiological factors as the Lasso score of GSRS-IBS total score. GSRS-IBS diarrhoea and GSRS-IBS satiety also showed significant (p < 0.05), but modest, correlations with their respective Lasso scores. After the false discovery rate (FDR) correction, a small number of correlations lost their significance. These associations were evenly distributed among the different neurophysiological examinations, as seen in Supplementary Table 1.

## Discussion

In this study, the correlations among IBS symptoms and a wide range of neurophysiological factors were explored to gain knowledge of the associations between symptoms and the complex pathophysiology of IBS and to extract the most relevant neurophysiology measures for GI symptom severity in IBS. Modest associations were seen among some of the different neurophysiological factors, and between some of the neurophysiological factors and GI symptoms. The associations between combinations of neurophysiological factors and GI symptoms were stronger, but the benefit of this approach relative to assessing the neurophysiological factors individually was modest, further highlighting the complexity of symptom generation in IBS.

In previous studies, some individual neurophysiological factors used in this study have been linked to elevated symptom severity, although to the best of our knowledge, GI motility, secretion, sensitivity and ANS and CNS function have not been analysed simultaneously in IBS patients before. Therefore, the associations among these factors are largely unknown, even though some previous studies have presented links between some of these factors. Rectal sensitivity has been found to be associated with rectal compliance^32^, the rectal tone response after meal intake^33^ and with baroreceptor sensitivity^34^. There is also a study that has demonstrated a link between rectal compliance and colonic transit time in IBS patients with urgency^35^. Therefore, due to the relative paucity of comprehensive and detailed neurophysiology assessment in IBS patients in the literature, the first set of analyses in this study explored associations between the different individual neurophysiological factors, as well as their association with IBS symptoms. The overall finding from these analyses showed associations between different measures within the same neurophysiological examination, e.g. between rectal sensory thresholds during balloon distensions, even though significant (p < 0.05), but weaker, associations were seen between measures from different neurophysiological measurements. When assessing the association with IBS symptom severity, modest associations between individual neurophysiology measures and symptom severity were noted, mainly confirming previous findings with associations between symptoms and visceral hypersensitivity, colonic transit and psychological distress in IBS^12,13,24,36^. Furthermore, the absence of associations with small intestinal motility and secretory measures, as well as rectal tone response and ANS variables, is noteworthy. The analysis approach used in this study is limited by the inability to articulate on the cause and effect between the different neurophysiological aberrancies and the severity of GI symptoms seen in the patient cohort, although it is well suited for the exploration of associations between measures.

Our hypothesis for the creation of the neurophysiology scores was that the neurophysiological factors could influence symptoms through different, but complimentary mechanisms, and therefore have additive effects on IBS symptoms. Furthermore, we hypothesized that even slight changes in the neurophysiological factors could lead to symptom generation through interactions. Based on these assumptions, we included all values deviating from the mean into the conjunct neurophysiology score in the analysis, creating a score where a value close to zero would indicate a normally functioning gut, including its interactions with ANS and CNS, and a high score would reflect many or high aberrant values in neurophysiological examinations as a proxy indicator of an abnormal gut-brain interaction. In previous studies from our group assessing individual neurophysiological factors included in this study, IBS patients have shown abnormalities in all of these factors relative to healthy controls, namely small intestinal motility^20^ small intestinal secretion^21^, ANS function^37^ colonic transit time^13^, rectal sensorimotor function^12,33^ and psychological distress^38^. For some, but not all of these, modest associations with IBS symptom severity were noted.

The variable reduced neurophysiology scores (Lasso scores) were found to correlate stronger with GI symptoms than the scores originating from all factors combined, i.e. the overall neurophysiology score. However, the gain was modest. There are several potential explanations for this. The limited range of the scales of the outcome measures (GSRS-IBS) might limit the level of detail in the results. The variable selection technique, Lasso regression, was chosen in accordance with previous studies^39,40^ to achieve an automatic variable selection. If the characteristics of the training set and the test set in the regression analysis are divergent, the variable selection might result in a suboptimal association with the whole cohort, i.e. the training and test sets combined. Furthermore, the Lasso regression has been described to occasionally choose the next-to-best combination of variables^41^ if there are more than one combination of variables with high predictive value. Lastly, as IBS has been proposed to consist of several diseases or subgroups with similar symptoms due to its heterogeneity^42,43^, a clearer association between GI symptoms and neurophysiological factors might arise when studying more homogenous subgroups of patients^44^. This way of extracting important factors for symptom generation in IBS, might be useful in future identification of new IBS subgroups, rather than subgrouping IBS patients solely based on the predominant bowel habit.

Two of the neurophysiological factors were present in every variable reduced neurophysiology score; the proxy measure of CNS function, i.e. psychological distress, and visceral sensitivity parameters, which implicate that they were the most important factors for the GI symptom pattern in this patient cohort. These two parameters were also seen to be central in the network analysis seen in Fig. 1, as they had the largest number of associations to other measures. The neurophysiology scores for GSRS-IBS total score and GSRS-IBS pain consisted of only these two factors, which is in accordance with previous studies, where these two pathophysiological factors have been shown to be of importance for symptom severity in IBS^12,24,38^. With this study, we have strengthened the view on psychological distress and visceral hypersensitivity as central factors for symptom generation in IBS. This is also in agreement with the findings from a recent publication from our group^36^, where a proportion of the subjects from this study was included (n = 137), in addition to patients from two other large patient cohorts. In that study, the focus was on visceral hypersensitivity, psychological distress and colonic transit, whereas the present study included a larger number of neurophysiological factors, albeit in a smaller cohort.

To provide a comprehensive model of the gut-brain axis, i.e. the bidirectional communication between the CNS, ANS and the enteric nervous system^45^, proxy measures of the function of the CNS and ANS were included among the neurophysiological variables, which may be viewed as a limitation. As the function of the ANS is only assessable through indirect measures such as heart rate variability^37,46^ or BR function, the BR sensitivity (BRS) was used in this study as a proxy measure of ANS function^14,15,34^, together with the BR effectiveness index, as it has been suggested to provide complementary information to the BRS^47^. The HAD scale is a widely used questionnaire assessing psychological distress, which has previously been used as a proxy measure for CNS function^48,49^, as we did in this study. Although it is widely used, it provides an incomplete measure of the whole function of the CNS.

To increase the validity or accuracy of the neurophysiology score, replacement or addition of certain neurophysiological factors could be discussed. The assessment of CNS dysfunction could be improved with addition of brain imaging, which could provide both structural^50^ and functional information about the CNS^51^. Furthermore, including heart rate variability and other ANS measures could strengthen the assessment of the ANS function^52^. Moreover, more detailed assessment of GI motor, sensory and secretory function at different levels in the GI tract could be considered. Lastly, addition of measures of gut microbiota composition and GI immune and barrier function^3^ to the most important factors for GI symptoms demonstrated in this study, could further broaden the pathophysiological assessment, potentially leading to a more precise prediction of GI symptoms in IBS patients. Although an addition of more variables would increase the risk of noise in the model, a computerized reduction of variables, such as the Lasso method used in this study, would nonetheless extract the relevant measures for GI symptom severity.

Another limitation of this study might be the choice of analysis methods, as association analyses are well suited for the exploration of correlations between measures, but is unfit for unravelling cause and effect between the different neurophysiological aberrancies and the severity of GI symptoms seen in the patient cohort.

To conclude, modest associations between GI symptoms, and individual as well as combinations of neurophysiological factors were seen in this study. The results from this study provides new insights into the complex interactions between symptom severity and neurophysiological measures in IBS. Further studies with an expanded range of neurophysiological variables of importance for symptom generation in IBS can be considered. However, inclusion of other factors putatively involved in the pathophysiology of IBS, such as the microbiome and GI immune and barrier function, in addition to the most important factors for symptom generation found in this study, might provide an even more complete picture of the associations between pathophysiological factors and GI symptoms in IBS.

## Methods

### Study participants

IBS patients, 18–65 years old, with IBS according to Rome II criteria^53^, were included for participation in a study assessing the relevance of various pathophysiological factors for IBS symptoms between the years of 2002–2007 in our outpatient clinic specialized in functional GI disorders at Sahlgrenska University hospital, Gothenburg, Sweden^12,13,33^. This study is a retrospective assessment of all the neurophysiology measures and GI symptom data available from this patient cohort. This cohort has been included in previous publications from our group, but with different research questions and/or focus on only a proportion of all the neurophysiology measurements included in the analyses in this study^12,13,21,24,33,36,37^. Most of the patients were referred to our unit from primary care. The diagnosis of IBS was confirmed by an experienced gastroenterologist (MS), and if considered necessary, additional investigations to rule out organic GI disorders were performed. Any medication with known effects on the GI system was discontinued prior to the investigations. The patients were subdivided into IBS subgroups, i.e. constipation-predominant IBS (IBS-C), diarrhoea-predominant IBS (IBS-D) or alternating IBS (IBS-A)^53^. Exclusion criteria were other gastrointestinal diseases explaining the abdominal symptoms, severe physical or psychiatric disease, or pregnancy. The Regional Ethical Review Board at the University of Gothenburg approved the study (Approval number S489-02) and all patients received oral and written information about the study and provided informed consent prior to inclusion. The study was carried out in accordance with the guidelines and regulations of the Declaration of Helsinki.

### Questionnaires

The patients completed the Gastrointestinal Symptom Rating Scale, IBS version (GSRS-IBS)^31^ for assessment of GI symptom severity. The Hospital Anxiety and Depression scale (HAD)^30^, which is a measure of psychological distress, e.g. anxiety and depression, in non-psychiatric patients, was also completed by the patients and in this study used as a proxy for CNS dysfunction. For details, see Supplementary material.

### Neurophysiology measures

#### Rectal sensorimotor function

Rectal sensitivity, and compliance, as well as the rectal tone response after meal intake were assessed during a rectal balloon distension protocol by using an electronic barostat (Dual Drive Barostat, Distender Series II; G&J Electronics). The pressure thresholds for the first sensation and pain during the balloon distensions in the fasting state were used in this study as measures of visceral sensitivity^12^. After the first sequence of distensions, the patients ingested a standardized meal, and the early (0–25 minutes) and late (25–50 minutes) rectal tone responses were calculated from the average change in percent in the rectal balloon volume at the operating pressure. The rectal static and dynamic compliance of the rectum were calculated from the pressure-volume curve during first five distension steps of the balloon distension sequence^33^. The details about this protocol can be found as Supplementary material.

#### Small bowel motility and secretion

After an overnight fast, the patients were transnasally intubated with a 8-channel multilumen polyvinyl tube (Arndorfer Inc., Greendale, WI, USA). The motility and secretion of the small bowel were examined with a jejunal manometry catheter, with saline liquid infusion acting as a flowing electrode, during a 3 h fasting recording and 1 h after ingestion of a standardized meal, as described in detail in the Supplementary material. The mean fasted and fed contraction frequencies from the motility recordings were used in the analyses, as well as the total phase III time, consisting of the total time with phase III activity in seconds during the three hours long examination period during fasting. Furthermore, the mean and maximum transmural potential difference, as well as the rate of rise of the potential difference were calculated, as measures of reactivity of secretomotor neurons (small intestinal secretion)^21^.

#### Autonomic nervous system function

The BR function, in this study used as a measure for ANS function^54^, was assessed through a simultaneous electrocardiography, arterial blood pressure and heart rate recording. The baroreceptor sensitivity and baroreceptor effectiveness index^29^, as described in detail in the Supplementary material, were used in the analysis of this study.

#### Colonic motility

Colonic transit time was used as an indirect measure of colonic motility and was measured using a technique with radio-opaque markers, as described in the Supplementary material. In this study, the overall colonic transit time in days was used for the analysis^13^.

### Data analysis and statistics

#### Descriptives

Demographic factors derived from the whole IBS cohort are described by medians with interquartile range in continuous variables, and numbers with percentages in categorical variables. The calculations were made in R (version 3.5.1 - “Feather Spray”). Chi squared tests or Kruskal-Wallis tests with two-tailed p-values were performed to compare medians between IBS subgroups. A p-value <0.05 was considered significant.

#### Correlations among neurophysiological factors and IBS symptoms

Analyses of correlations between the GSRS-IBS total score and the domain scores of GSRS-IBS (pain, bloating, constipation, diarrhoea and satiety), and the neurophysiological factors (rectal sensorimotor function, small bowel motility and secretion, ANS and CNS function, and colonic motility) were calculated with Spearman’s correlation. For this we used the cor function in the stats package in R. The analyses were visualized, uncorrected for multiple comparisons, through a correlation network made by the qgraph function in the qgraph package in R. Two-tailed p-values for the correlations were calculated through the corr.test function in the psych package in R. A p-value <0.05 was considered significant.

#### Multivariate analyses

Multivariate analyses were performed in order to determine if a combination of neurophysiological factors (neurophysiology score) would show stronger associations with the GI symptom severity than the individual neurophysiological factors. As presented below, two different processes, one with variable selection and one without, were used for calculation of neurophysiology scores, which were then analysed regarding their associations with IBS symptom severity.

### Preprocessing of data

The patients who had completed the outcome variables GSRS-IBS were included in the multivariate analyses. The missing values of the dataset were imputed by multiple imputation, as implemented in missForest package in R.

#### Overall neurophysiology score

A neurophysiology score was created, using all of the 16 different neurophysiologic factors (overall neurophysiology score) in each patient. The neurophysiologic factors were first standardized by the mean (z-scores) and then individually processed. The aim of the processing step was to create a neurophysiology score where a normally functioning gut would be reflected with a score close to zero, whereas many or high aberrancies would give a high total score. All values that were different from the mean in each parameter added to or subtracted from the overall neurophysiology score, as seen in Table 4. Specifically, values considered as abnormal, e.g. a low pain threshold, increased the overall neurophysiology score, whereas values, which were considered normal, e.g. a low value of psychological distress or a high rectal pain threshold, decreased the overall neurophysiology score. After the processing step, the processed values of all of the neurophysiological factors were added up in each patient, resulting in an overall neurophysiology score.

**Table 4:** Contribution of the neurophysiological factors to the overall neurophysiology score.

Neurophysiologic factor	Low values	High values
HAD	−	+
Colonic transit time	+	+
Early rectal tone response	+	+
Late rectal tone response	+	+
Rectal dynamic compliance	+	+
Rectal static compliance	+	+
First sensation threshold	+	−
Pain threshold	+	−
SI phase III time	+	+
SI fasted contraction frequency	+	+
SI fed contraction frequency	+	+
SI potential difference, mean	−	+
SI potential difference, max	−	+
SI potential difference, rate of rise	−	+
Baroreceptor sensitivity	+	−
Baroreceptor effectiveness	+	−

+:Increases the neurophysiology score; −: decreases the neurophysiology score; HAD: Hospital Anxiety and Depression scale; High values: >0 in z-score; Low values: <0 in z-score; SI: small intestine/intestinal.

#### Neurophysiology score using variable selection

In addition to the overall neurophysiology score described above, scores consisting of selected variables were also calculated for each of the GSRS-IBS domains and the total score, in order to see if this is process would lead to stronger associations with the GI symptom severity measures. The Least Absolute Selection and Shrinkage Operator regression model (Lasso) was used as the variable selection method^39,40^. The six neurophysiology scores consisting of selected measures for each of the GI symptom severity measures (GSRS-IBS total score and the five GSRS-IBS domain scores) are called ‘Lasso scores’ throughout this manuscript. The Lasso regression analysis is described in detail in the Supplementary material.

### Correlations between neurophysiology scores and IBS symptoms

As the last step, Spearman’s correlations were calculated between the domains and total score of GSRS-IBS, and all single neurophysiological factors, and the neurophysiology scores (i.e. the overall neurophysiology score and the Lasso scores). The correlations were done with the cor function in the stats package and were illustrated as a heatmap, made by the heatmap.2 function in the gplots package in R, and a barplot. Two-tailed p-values for the correlations (p < 0.05) were calculated by the cor.test function in the stats package, and were adjusted for multiple comparisons with false discovery rate (FDR) adjustment. The heatmap illustrated the correlations between the domains and total score of GSRS-IBS, and the single neurophysiological factors, the overall neurophysiology score and the Lasso scores. The barplot compared the correlations between the domains and total score of GSRS-IBS and the overall neurophysiology score vs. the Lasso scores.

## Supplementary information

Supplemental information.

## Data Availability

The datasets generated during and/or analysed during the current study are available from the corresponding author upon request.
