# Supplementary material for: Associations among neurophysiology measures in irritable bowel syndrome (IBS) and their relevance for IBS symptoms
Source: Sci Rep. 2020 Jun 17;10:9794. doi: 10.1038/s41598-020-66558-w (PMC7300023; doi:10.1038/s41598-020-66558-w)
Supplement: Supplementary file 1 — Supplemental information. [file 41598_2020_66558_MOESM1_ESM.doc]

**Associations among neurophysiology measures in irritable bowel syndrome (IBS) and their relevance for IBS symptoms.**

**Authors**

Irina Midenfjord^1^, Annikka Polster^1^, Henrik Sjövall^1^, Peter Friberg^2^, Hans Törnblom^1^, Magnus Simrén^1,3*^

1. Department of Internal Medicine and Clinical Nutrition, Institute of Medicine, Sahlgrenska Academy, University of Gothenburg, Gothenburg, Sweden.
2. Department of Molecular and Clinical Medicine, Sahlgrenska Academy at Gothenburg University, Gothenburg, Sweden.
3. Centre for Functional GI and Motility Disorders, University of North Carolina, Chapel Hill, NC, United States.

Address for correspondence

Prof. Magnus Simrén, MD, PhD

Department of Internal Medicine and Clinical Nutrition

Institute of Medicine

Sahlgrenska Academy, University of Gothenburg

40530 Gothenburg, Sweden.

e-mail: magnus.simren@medicine.gu.se

fax number: +46317412917

telephone number: +46313421000

**Supplementary Material**

**Methods**

Questionnaires

*Gastrointestinal Symptom Rating Scale*

The Gastrointestinal Symptom Rating Scale, IBS version, (GSRS-IBS) rates IBS symptom severity ^1^. The questionnaire consists of 13 questions using a 7-point Likert scale. These are combined into five domains: Abdominal pain syndrome (pain), Bloating syndrome (bloating), Constipation syndrome (constipation), Diarrhea syndrome (diarrhea) and Satiety syndrome (satiety). In addition to the five domains, a total score can be obtained from the questionnaire. A higher score means a higher IBS symptom severity.

*Hospital Anxiety and Depression scale*

Scores for anxiety and depression were measured by the Hospital Anxiety and Depression scale (HAD) ^2^. It is a 14-item questionnaire, intended to be used in non-psychiatric populations, where half of the questions address anxiety and the other half address depression. The total score of HAD, i.e. including both subscales, is used as a measure for overall psychological distress, and serves as a proxy measure for central nervous system function in this study. The maximum total score is 42, and the maximum score of the two subscales are 21, respectively. A higher score indicates more severe psychological distress. To define patients with and without anxiety or depression, the validated cut-offs of ≥8 ^3^ were used in the two subscales, respectively, which defines subjects with possible and probable anxiety and depression.

Neurophysiological measures

*Rectal sensorimotor function*

The examination of rectal sensorimotor function (rectal visceral sensitivity, rectal tone and rectal compliance) was performed with a distension protocol with rectal isobaric phasic balloon distensions by an electronic barostat (Dual Drive Barostat, Distender Series II; G&J Electronics) before and after a standardized meal. In the fasting state, phasic distensions lasting 30 sec were performed, with 30 sec rest periods between the distensions with the balloon pressure at the operating pressure (minimal distending pressure + 2mmHg). The distensions were performed with stepwise increments of 5 mmHg until pain was reported or a pressure of 70 mmHg was reached. The thresholds for rectal fullness (first sensation), urge to defecate, discomfort and pain were determined during the distensions, as described in detail by Törnblom et al ^4^. The thresholds for first sensation and pain were used in this study as measures of visceral sensitivity. During the distension sequence in the fasted state, the static compliance was calculated as the mean of the volume/pressure relationship, whereas the dynamic compliance was measured as change in volume per change in pressure, in the linear part of the slope of the compliance curve, i.e. from the first five distension steps ^5^. Thereafter, the patients ingested a standardized meal with the balloon pressure at the operating pressure, as previously described ^4^. The meal consisted of a 560 mL, 800 kcal liquid meal (60% fat, 20% carbohydrate, 20% protein; 100 mL cream, 85 mL water, 100 mL Nutridrink, 275 mL Fortimel (Nutricia Nordic AB, Stockholm, Sweden)) ^6^. The early (0―25 minutes) and late (25―50 minutes) rectal tone responses were calculated from the average change in percent (mean of every 5 minutes) in the rectal volume after the ingestion of the standardized meal, relative to the balloon volume before meal intake.

*Small bowel motility and secretion*

After an overnight fast, the patients were transnasally intubated with a 8-channel multilumen polyvinyl tube (Arndorfer Inc., Greendale, WI, USA) and the motility and secretion of the small bowel were examined, as thoroughly described by Mellander et al ^7^. Six of the channels were used for assessment of motility and these were placed in the proximal jenunum, one at the duodenojejunal junction, three in the papilla region of the mid duodenum at a distance of 1.5 cm between each channel, and one in the antrum ^8^. The catheter was placed during fluoroscopy and the pressure-recording channels were perfused with water at a rate of 30 mL/h by a narrow capillary pneumohydraulic perfusion system (No 6009; Triplus, Kungsbacka, Sweden), which was connected to the pressure transducer (23 DC; Statham Instruments Oxnard, CA, USA). The small intestinal transmural potential difference (PD), reflecting mainly electrogenic chloride secretion, was measured between calomel half cells (Radiometer, Copenhagen, Denmark) at the recording points in the proximal duodenum and the jejunum using an infusion of isotonic saline (instead of water) as a flowing electrode. The reference electrode was inserted subcutaneously into the left forearm and consisted of a saline filled plastic cannula (Venflon; outer diameter 1.0 mm; Viggo-Spectramed, Helsingborg, Sweden). This system enabled the measurement of pressure and PD at the same site. A software program filtered and processed the voltage signal by a high impedance amplifier and calculated mean values for intraluminal pressure and transmural PD (Polygram 5; Synectics Medical, Stockholm, Sweden). The raw data files were stored as ASCII files and were data processed in Matlab (The Mathworks, Lowell, MA, USA; Release 14SP3).

The small bowel motility and transmucosal potential difference was recorded for three hours in the interdigestive state and for one hour after a standard meal consisting of oat porridge (1 dl oatmeal, 2 dl water) with milk (2 dl), apple sauce (2 tbsp) and one sandwich with hard cheese, 513 kcal (34% fat, 52% carbohydrates, 14% protein).

Fasted and fed contraction frequencies were calculated from the motility recordings described above. For the fasted contraction frequencies, the time period preceding the first phase III period by 17 minutes and ending 32 minutes after the end of phase III was analyzed in each study participant, as previously described ^8^, and the mean value was used in the data analysis. For the fed contraction frequencies, the first hour following the meal intake was analyzed. The total phase III time consisted of the total period with phase III activity in seconds during the three hour long examination period. The data was then low-pass filtered and resampled from 4 Hz to 0.2 Hz using the idresamp function in Matlab (The Mathworks, Lowell, MA, USA; Release 14SP3) to avoid effects of individual contractions. The mean PD during late phase II to the beginning of phase III was calculated, as was the maximum PD during phase III and the rate of rise (mV s^-1^) of PD in the initial phase III.

*Autonomic nervous system function*

The examination took place after an over-night fast. The baroreceptor sensitivity (BRS) and effectiveness index (BEI), proxies for the autonomic nervous system (ANS) function, were calculated during electrocardiography with simultaneous continuous arterial blood pressure and heart rate recording through a finger cuff (Finapres, TNO, Amsterdam, The Netherlands), which was attached to the middle finger on the participant’s right hand. After a ten minutes long adaption/habituation period, BRS and BEI were calculated during metronome respiration (12 inhalations/minute) to avoid the impact from spontaneous breathing on the pulse and blood pressure. BRS was calculated as the change in interbeat intervals (milliseconds) in the low-frequency band (0.04–0.15 Hz) when the arterial blood pressure changed 1 mmHg ^9^. BEI was analyzed as the ratio between the total number of detected baroreceptor sensitivity sequences (sequences where the interbeat interval and blood pressure monotonously increase or decrease ^9^) and the total number of blood pressure ramps (threshold ≥1 mmHg) during a given timeframe ^10^.

*Colonic motility*

The colonic transit time was extracted through counting of ingested radio-opaque markers (ROM) (Transit-Pellets®, Medifactia AB, Stockholm, Sweden) ^11,12^. Ten ring-formed ROMs were ingested each day at 08.00 for five consecutive days. On the sixth day, five rod formed ROMS were ingested at 08.00 and five at 20.00, for more precise transit time measurement for patients with fast colonic transit. On the following morning at 08.00, the ROMs remaining in the colon were counted during fluoroscopy (Arcadis Avantic VC 10 A, Siemens, München, Germany). The counted ROMs were then divided by the daily intake of ROMs (i.e. 10) to achieve the colonic transit time in days.

*Neurophysiology score using variable selection (LASSO).*

The Least Absolute Selection and Shrinkage Operator regression method (Lasso) was used as variable selection method for each GSRS-IBS domain and the GSRS-IBS total score, with the purpose to define a less complex model. The method performs feature elimination through shrinkage of the regression coefficients/slope of the regression lines of simple regressions, which reduces variance, shrinks the coefficient estimates toward zero, and thereby leads to automatic variable selection. The shrinkage is expressed by the parameter λ. A λ of zero performs no shrinkage to the variable coefficients, and a higher λ equals a larger shrinkage of the coefficients, with increasing number of eliminated variables. The purpose of the shrinkage is to penalize model complexity and to avoid overfitting.

In the first step of the Lasso variable selection process, the λ with the lowest root mean squared error (RMSE), i.e. the closest fit of the model, was selected through a 1000 times calculation on the training set, consisting of 70% of the total cohort, through the cv.glmnet and glmnet functions in the glmnet package in R. Then, the Lasso regression was performed through 10-folded cross-validation by cv.glmnet, glmnet and predict.glmnet functions in the glmnet package in R, with the λ obtained from the previous step, and with α set to 1 to obtain Lasso regression. The RMSE was extracted as a measure of fit of the model. This process lead to the identification of the factors most strongly associated with the outcome variables, i.e. the GSRS-IBS variables.

**References**

1. Wiklund I, Fullerton S, Hawkey C., et al. An Irritable Bowel Syndrome-Specific Symptom Questionnaire: Development and Validation. *Scand J Gastroenterol*. 2003;38(9):947-954.

2. Zigmond AS, Snaith RP. The hospital anxiety and depression scale. *Acta Psychiatr Scand*. 1983;67(6):361-70.

3. Bjelland I, Dahl AA, Haug TT, Neckelmann D. The validity of the Hospital Anxiety and Depression Scale. *J Psychosom Res*. 2002;52(2):69-77.

4. Törnblom H, Van Oudenhove L, Tack J, Simrén M. Interaction between preprandial and postprandial rectal sensory and motor abnormalities in IBS. *Gut*. 2014;63(9):1441-1449.

5. Floyd BNI, Camilleri M, Andresen V, Esfandyari T, Busciglio I, Zinsmeister AR. Comparison of mathematical methods for calculating colonic compliance in humans: Power exponential, computer-based and manual linear interpolation models. *Neurogastroenterol Motil*. 2008;20(4):330-335.

6. Simrén M, Agerforz P, Björnsson ES, Abrahamsson H. Nutrient-dependent enhancement of rectal sensitivity in irritable bowel syndrome (IBS). *Neurogastroenterol Motil*. 2007;19(1):20-29.

7. Mellander A, Järbur K, Sjövall H. Pressure and frequency dependent linkage between motility and epithelial secretion in human proximal small intestine. *Gut*. 2000;46(3):376-384.

8. Larsson MH, Simrén M, Thomas EA, Bornstein JC, Lindström E, Sjövall H. Elevated motility-related transmucosal potential difference in the upper small intestine in the irritable bowel syndrome. *Neurogastroenterol Motil*. 2007;19(10):812-820.

9. Swenne CA. Baroreflex sensitivity: Mechanisms and measurement. *Netherlands Hear J*. 2013;21(2):58-60.

10. Di Rienzo M, Parati G, Castiglioni P, Tordi R, Mancia G, Pedotti A. Baroreflex effectiveness index: an additional measure of baroreflex control of heart rate in daily life. *Am J Physiol Integr Comp Physiol*. 2001;280(3):R744-R751.

11. Sadik R, Björnsson E, Simrén M. The relationship between symptoms, body mass index, gastrointestinal transit and stool frequency in patients with irritable bowel syndrome. *Eur J Gastroenterol Hepatol*. 2010;22(1):102-108.

12. Törnblom H, Van Oudenhove L, Sadik R, Abrahamsson H, Tack J, Simrén M. Colonic transit time and IBS symptoms: What’s the link? *Am J Gastroenterol*. 2012;107(5):754-760.

**Supplementary Table 1:** Correlations between domains and total score of GSRS-IBS, and single neurophysiological factors, the overall neurophysiology score, or the Lasso scores.

| **GSRS-IBS domain** | **Pain** | | **Bloating** | | **Constipation** | | **Diarrhea** | | **Satiety** | | **Total score** | |
| --- | --- | --- | --- | --- | --- | --- | --- | --- | --- | --- | --- | --- |
|  | Rho | P-value | Rho | P-value | Rho | P-value | Rho | P-value | Rho | P-value | Rho | P-value |
| **HAD** | **0.22** | **0.006** | **0.17** | **0.03** | **0.24** | **0.005** | 0.17 | 0.05 | **0.20** | **0.02** | **0.28** | **<0.001** |
| **Colonic transit time** | **-0.19** | **0.02** | -0.02 | 0.79 | **0.33** | **<0.001** | **-0.3** | **>0.001** | 0.04 | 0.68 | -0.07 | 0.40 |
| **Early rectal tone response** | -0.07 | 0.47 | 0.03 | 0.78 | 0.08 | 0.41 | -0.05 | 0.69 | 0.06 | 0.56 | 0.001 | 0.98 |
| **Late rectal tone response** | -0.08 | 0.46 | -0.02 | 0.78 | 0.07 | 0.46 | -0.08 | 0.44 | 0.03 | 0.70 | -0.04 | 0.65 |
| **Rectal dynamic compliance** | 0.01 | 0.91 | **0.21** | **0.008** | 0.12 | 0.16 | -0.01 | 0.97 | 0.04 | 0.65 | 0.12 | 0.15 |
| **Rectal static compliance** | -0.02 | 0.81 | 0.15 | 0.06 | 0.02 | 0.79 | -0.13 | 0.14 | 0.05 | 0.61 | 0.02 | 0.81 |
| **Rectal first sensation threshold** | -0.12 | 0.18 | **-0.19** | **0.02** | 0.05 | 0.60 | -0.13 | 0.15 | -0.14 | 0.11 | -0.16 | 0.04 |
| **Rectal pain threshold** | **-0.30** | **<0.001** | **-0.20** | **0.01** | -0.06 | 0.49 | **-0.26** | **0.001** | **-0.22** | **0.01** | **-0.31** | **<0.001** |
| **SI phase III time** | -0.01 | 0.91 | -0.11 | 0.19 | -0.01 | 0.86 | 0.06 | 0.63 | -0.01 | 0.87 | -0.01 | 0.92 |
| **SI fast. contraction frequency** | -0.07 | 0.46 | **-0.21** | **0.01** | -0.11 | 0.20 | -0.02 | 0.97 | -0.14 | 0.11 | **-0.18** | **0.03** |
| **SI fed contraction frequency** | 0.06 | 0.51 | -0.04 | 0.68 | 0.05 | 0.58 | 0.01 | 0.97 | 0.06 | 0.56 | 0.04 | 0.71 |
| **SI PD mean** | 0.10 | 0.29 | **0.23** | **0.005** | **0.19** | **0.02** | 0.03 | 0.87 | 0.16 | 0.07 | **0.20** | **0.01** |
| **SI PD max** | 0.03 | 0.78 | 0.08 | 0.32 | 0.20 | 0.02 | -0.01 | 0.97 | 0.14 | 0.11 | 0.11 | 0.15 |
| **SI PD rate** | 0.07 | 0.46 | 0.10 | 0.22 | 0.04 | 0.64 | **0.18** | **0.03** | -0.02 | 0.79 | 0.14 | 0.07 |
| **BR sensitivity** | 0.08 | 0.46 | **0.24** | **0.003** | **0.28** | **0.001** | 0.03 | 0.85 | 0.13 | 0.12 | **0.23** | **0.003** |
| **BR effectiveness** | -0.06 | 0.52 | -0.15 | 0.05 | **-0.18** | **0.03** | 0.002 | 0.98 | -0.14 | 0.11 | -0.14 | 0.07 |
| **Overall neuro-physiology score** | **0.21** | **0.008** | **0.23** | **0.005** | 0.10 | 0.26 | 0.16 | 0.06 | 0.13 | 0.12 | **0.25** | **0.001** |
| **Lasso pain** | **0.38** | **<0.001** | **0.27** | **0.001** | **0.20** | **0.02** | **0.27** | **0.001** | **0.30** | **<0.001** | **0.4** | **<0.001** |
| **Lasso bloating** | **0.3** | **<0.001** | **0.20** | **0.01** | 0.11 | 0.21 | 0.21 | 0.01 | **0.20** | **0.02** | **0.29** | **<0.001** |
| **Lasso constipation** | **0.18** | **0.03** | **0.17** | **0.03** | 0.14 | 0.12 | 0.08 | 0.43 | 0.09 | 0.29 | **0.20** | **0.01** |
| **Lasso diarrhea** | **0.33** | **<0.001** | **0.24** | **0.003** | 0.15 | 0.07 | **0.29** | **<0.001** | **0.25** | **0.002** | **0.37** | **<0.001** |
| **Lasso satiety** | **0.35** | **<0.001** | **0.33** | **<0.001** | **0.26** | **0.002** | **0.18** | **0.03** | **0.29** | **<0.001** | **0.40** | **<0.001** |
| **Lasso total** | **0.38** | **<0.001** | **0.27** | **0.001** | **0.20** | **0.02** | **0.27** | **0.001** | **0.30** | **<0.001** | **0.40** | **<0.001** |

Correlations are calculated as Spearman’s rho, with correction for false discovery rate (FDR). Significant correlations are marked in bold . The correlations that lost significance after FDR correction are presented in red.

BR: baroreceptor; GSRS-IBS: Gastrointestinal Symptom Rating Scale, IBS version; HAD: Hospital Anxiety and Depression scale; Lasso: Least Absolute Selection and Shrinkage Operator regression-derived neurophysiology scores; Overall score: Neurophysiology scores derived from all 16 neurophysiological factors; PD: potential difference; SI: small intestine/intestinal.
